# *Ninjurin 2* rs118050317 gene polymorphism and endometrial cancer risk

**DOI:** 10.1186/s12935-020-01646-5

**Published:** 2021-01-04

**Authors:** Yimin Cheng, Liting Yang, Guangyao Shi, Peng Chen, Liang Li, Hangrong Fang, Chao Chen

**Affiliations:** 1 The National Engineering Research Centre for Miniaturized Detection Systems, College of Life Science, Northwest University, #229 North TaiBai Road, Xi’an, 710069 Shaanxi China; 2 The Hospital of Xi’an Shiyou University, Xi’an, People’s Republic of China; 3 Department of Radiotherapy, Shaanxi Provincial Tumor Hospital, Xi’an, People’s Republic of China; 4 Department of Pathology, Xi’an No.3 Hospital, The Affiliated Hospital of Northwest University, Xi’an, People’s Republic of China

**Keywords:** Endometrial cancer, *Ninjurin2*, Clinical index, Single nucleotide polymorphisms

## Abstract

**Background:**

Endometrial cancer is one of the most common female reproductive system tumors. *Ninjurin2* (*NINJ2*) is a new adhesion factor. As a vascular susceptibility gene, it is highly expressed in other cancers and promotes the growth of cancer cells. We conducted an association analysis between *NINJ2* gene polymorphism and endometrial cancer risk.

**Methods:**

Five SNPs rs118050317, rs75750647, rs7307242, rs10849390 and rs11610368 of *NINJ2* gene were genotyped in 351 endometrial cancer patients and 344 healthy controls. The clinical index difference between cases and controls were tested by one-way analysis of variance. The allele and genotype frequency of cases and controls were been compared by Chi square test. The odds ratios (OR) with 95% confidence interval (95% CI) were examined by logistic regression analysis.

**Results:**

The SNP rs118050317 mutant allele C and homozygote CC genotype were significant increased the endometrial cancer risk (OR 1.46, 95% CI 1.04–2.06, *p* = 0.028; OR 8.43, 95% CI 1.05–67.89, *p* = 0.045). In the clinical index analysis, there were significant higher quantities of CEA, CA125 and AFP in cases serum than controls.

**Conclusion:**

The *NINJ2* gene polymorphism loci rs118050317 mutant allele C was associated with an increased risk of endometrial cancer. CEA, CA125 and AFP quantities were significant higher in endometrial cancer patients.

## Introduction

Endometrial cancer is malignant tumor of endometrium epithelial, which also is one of the most common female reproductive system tumors [1]. In historically, endometrial cancer was been classified as type I (80–90%) and type II (10-20%), in histopathological classification divided tumors into endometrioid and non-endometrioid [2, 3]. With the development of society and the improvement of economic conditions, the incidence of endometrial cancer has also increased year by year in China [4]. Increased estrogen exposure is a major risk factor for endometrial cancer, early menarche, infertility, obesity and late menopause are associated with endometrial cancer risk, too [5]. Moreover, women with a family history of endometrial cancer have a higher risk of the disease than those without a family history, suggesting that inherited genetic factors have an impact on the incidence of endometrial cancer [6].

In the genome-wide association study of endometrial cancer, the susceptibility locus close to *HNF1B* on chromosome 17q was associated with endometrial cancer [7]. Vivo et al. replicated previously identified associations with genetic markers located at 17q12 (rs4430796) near the *HNF1B* locus [8]. Five endometrial cancer risk SNPs loci on 13q22.1, 6q22.31, 8q24.21, 15q15.1 and 14q32.33 were identified by genome-wide association study [9, 10]. Moreover, the SNP locus in *ADIPOQ*, *MDM2*, *TP53* and leptin gene were associated with endometrial cancer, too [5, 11, 12], respectively. Despite there were a lot of endometrial cancer studies, the endometrium carcinogenesis remains poorly understood.

**Fig. 1:**
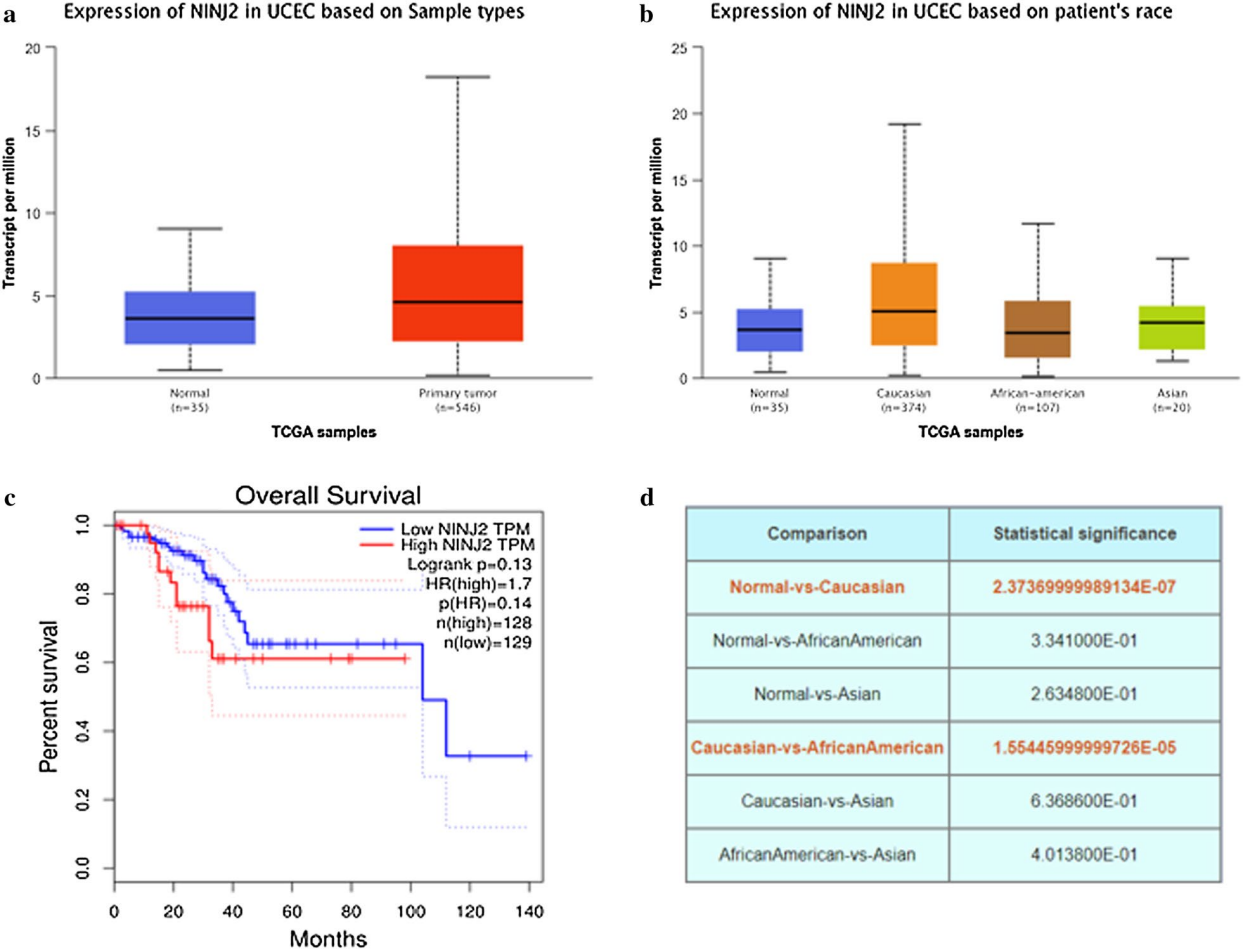
Expression of NINJ2 in endometrial carcinoma. **a** The UALCAN database predicts the expression of NINJ2 in normal and cancer populations. **b** The UALCAN database predicts the expression of NINJ2 in patient’s race. **c** The GEPIA database predicts the prognosis of NINJ2 in patients. **d** The P value of the box diagram in Figure B

*Ninjurin2* (*NINJ2*) is a transmembrane protein that mediates cell-to-cell and cell-to-extracellular matrix interactions during development, differentiation, and regeneration of nervous system, the genetic polymorphisms of *NINJ2* were associated with a decreased risk of Alzheimer’s disease [13], ischemic stroke [14] and large artery atherosclerotic stroke [15]. Although there were significant association between *NINJ2* gene polymorphisms and nervous system disease, but few studies were conducted between *NINJ2* gene polymorphisms and cancer (Fig. 1).

The chronic inflammation may play an important role in endometrial carcinogenesis. IL-6 gene had an association with endometrial cancer risk [16]. *NINJ2* can regulate the expression of IL-6 in human vascular endothelial cells. In this study we investigated whether alterations in the *NINJ2* gene can influence the risk of endometrial cancer. We performed the case control study to investigate the relationship between five SNPs (rs118050317, rs75750647, rs7307242, rs10849390 and rs11610368) of *NINJ2* gene and endometrial cancer risk in Chinese women. We take A as the allele of mutant, then, the dominant model is: AA + AG compared with GG. The recessive model was: AA compared with AG + GG. The co-dominant model was: AA, AG and GG were compared. The additive model is: AA ratio GG. We used these four genetic models to study the relationship between SNPs of different genotypes and clinical indicators in patients with endometrial cancer.

## Materials and methods

### Study population

351 endometrial cancer patients (55.66 ± 8.46) were diagnosed and selected from Shaanxi Provincial Tumor Hospital. All patients were clinical diagnosed as endometrial cancer by pathology. All cases have no radiotherapy, chemotherapy and no other cancer history. Among them, 184 were smaller than 55 years old and 167 were greater than 55 years old. 344 healthy controls (55.60 ± 8.43) were selected from the physical examination center of Tang du physical examination center. All controls have no history of cancer and age matched with cases. Among them, 178 were smaller than 55 years old and 166 were greater than 55 years old (Table 1).

**Table 1:** The basic information of cases and controls

Variable	Cases (n = 351)	Controls (n = 344)	*p*
Age (Mean ± SD)	55.66 ± 8.46	55.60 ± 8.43	0.923
≤ 55	184	178	
> 55	167	166	
Clinical index			
CEA	305	105	
Quantity in serum(ng/ml)	12.55 ± 3.95	2.24 ± 3.14	< 0.001*
CA125	299	105	
Quantity in serum (U/ml)	24.23 ± 56.30	13.25 ± 11.52	0.048*
CA199	285	95	
Quantity in serum (U/ml)	21.21 ± 58.23	13.91 ± 11.62	0.226
AFP	303	105	
Quantity in serum (ng/ml)	9.75 ± 5.28	2.77 ± 0.97	< 0.001*

*CEA* carcinoembryonic antigen, *CA* carbohydrate antigen, *AFP* alpha fetoprotein

*p < 0.05 indicates statistical significance

We collected clinical indicators of participants as much as possible, including Carcinoembryonic antigen (CEA), Carbohydrate antigen 125 (CA125), CA199, Human epididymis protein 4 (HE4), Alpha fetoprotein (AFP), Serum ferritin (SF) and Tumor necrosis factor (TNF). These clinical indicators are female tumor markers, and their dynamic changes are related to the disease, which can provide an important basis for the judgment, prognosis and treatment of the disease [17, 18]. All participators provided informed consent and the Ethical Committee approved by the study.

### SNP selection and genotyping

Five SNPs rs118050317, rs75750647, rs7307242, rs10849390 and rs11610368 were selected from *NINJ2* gene based on the minor allele frequency was greater than 0.05 in global population. The whole genome DNA was extracted from 5 ml peripheral blood using GoldMag-Mini whole blood genomic DNA purification kit (GoldMag Co. Ltd. Xi’an City, China). The primers of genotyping were designed by Agena on-line software (https://agenacx.com/online-tools/), the primer of this study were list in Table 2. Agena MassARRAY platform was used to detect the five SNPs in case and control group (Agena Bioscience, SanDiego, CA, USA).

**Table 2:** Primers used in this study

SNPs	2nd-PCRP	1st-PCRP	UEP_SEQ
rs118050317	ACGTTGGATGGTGTAGTGATTGACACCTG	ACGTTGGATGACAGGAGCTGGTCATGTTGC	ggggtGCCGATGGGGAAGGATTAG
rs75750647	ACGTTGGATGTGTTCGCTGTGTACTGGATG	ACGTTGGATGCCCCCACAAAATTACAAACC	CTAAAGCAGGGTGGAG
rs7307242	ACGTTGGATGAAATGCTTCTCCTGGAAGTC	ACGTTGGATGGCCCTAGCCTGTTTCTTTAG	CCAGATCACTAGCTCTGA
rs10849390	ACGTTGGATGTCACACAATCTCACAGGGAC	ACGTTGGATGGAAATCAGTACTGCCTGTGC	tcgccCAGGGACAGCCCGCTGCC
rs11610368	ACGTTGGATGTCTGTGACTCCTTGCCAATG	ACGTTGGATGCTCTGCAATGTTACACAGCC	CCTTGCCAATGGATAGAATAGAA

*UEP SEQ* unextended mini-sequencing primer

### Statistical analysis

The clinical index quantity in serum difference was tested by one-way analysis of variance by SPSS16.0 [19], see the following formula: (1) Establish test hypothesis and determine test level: H0: There was no difference between the two groups; H1: There are differences between the two groups, or they are not all equal; (2) Calculate the value of statistic F: SS_1_ is the mean sum of squares between the groups; SS_2_ is the sum of mean squares within the group. N is the number of groups. MS_1_ is between the groups; MS_2_ is within the group.

$${\text{MS}}_{ 1} \, = \,{\text{SS}}_{ 1} /{\text{N}} - 1$$

$${\text{MS}}_{ 2} \, = \,{\text{SS}}_{ 2} /{\text{DATA}} - {\text{N}}$$

$${\text{F}}\, = \,{\text{MS}}_{ 1} /{\text{MS}}_{ 2}$$

(3) Determine P value and make statistical inference. The Hardy–Weinberg equilibrium (HWE) *p* value of each SNP in controls was calculated by exact test, *p* value greater than 0.05 means the sample have reached a genetic balance. The formula of Fisher’s Exact test is as follows: p = (a + b)!(c + d)!(a + c)!(b + d)!/(a!b!c!d!n!). a, b, c, d, are the data in the contingency table, respectively.

The allele and genotype distribution in cases and controls were compared by Chi square test. The odds ratios (OR) with 95% confidence interval (95% CI) were examined by logistic regression analysis before and after adjustment for age [20]. Suppose the dependent variable Z and a set of independent variables x1, x2, x3,…, xn, where Z is a continuous variable, we can fit a linear equation:

$${\text{p}}({\text{y}}\, = \, 1)\, = \,{\text{ez}}/( 1\, + \,{\text{ez}}),$$

$${\text{p}}({\text{y}}\, = \,0)\, = \, 1/( 1\, + \,{\text{ez}}),{\text{ Z}}\, = \,\beta 0\, + \,\beta { 1}*{\text{x1}}\, + \,\beta { 2}*{\text{x2}}\, + \,\beta { 3}*{\text{x3 }} + \cdots \, + \,\beta {\text{ n }}*{\text{ xn}}$$

The least square method is used to estimate the value of each β coefficient.

odds = p/(1 − p), OR: Event occurrence probability of the experimental group (odds1)/Occurrence rate of events in control group (odds2).

The linkage disequilibrium and haplotype construction were analyzed by Haploview v4.2 [21].

## Results

In this study, we recruited 351 endometrial cancer patients and 344 healthy controls, the age of cases and controls were matched (*p *= 0.923). There were significant differences in CEA, CA125 and AFP quantity in serum between cases and controls (*p *< 0.001; *p *= 0.048; *p *< 0.001). All of three clinical indicators were higher quantity in cases than controls (Table 1).

In Table 3, we listed the basic information of the SNPs which including the chromosome, position, minor and common allele, the HWE *p* value of all SNPs were greater than 0.05, we calculated the minor allele frequency (MAF) of cases and controls. We found compared with common G allele, the rs118050317 C allele were significant increased 0.46-fold endometrial cancer risk (OR 1.46, 95% CI 1.04–2.06, *p* = 0.028).

**Table 3:** Basic information of candidate SNPs in this study

SNPs	Chromosome	Gene	Position	Minor allele	Common allele	HWE *p* value	MAF		OR (95% CI)	*p*
							Case	Control		
rs118050317	12	*NINJ2*	634,980	C	G	0.336	0.129	0.092	1.46 (1.04–2.06)	0.028*
rs75750647	12	*NINJ2*	638,831	A	G	0.617	0.333	0.314	1.09 (0.87–1.37)	0.440
rs7307242	12	*NINJ2*	641,529	A	T	0.197	0.138	0.149	0.92 (0.68–1.24)	0.576
rs10849390	12	*NINJ2*	646,086	G	A	0.638	0.372	0.360	1.05 (0.85–1.31)	0.646
rs11610368	12	*NINJ2*	662,624	A	G	0.053	0.127	0.130	0.97 (0.71–1.33)	0.869

*SNP* single nucleotide polymorphisms, *HWE* Hardy–Weinberg equilibrium, *MAF* minor allele frequency, *OR* odds ratio, *95% CI* 95% confidential interval

*p < 0.05 indicates statistical significance

In Table 4, we calculated the genotype distribution in cases and controls under four different genotype model (co-dominant, dominant, recessive and log-additive) before and after adjustment the age. After adjusted the age, compared with GG genotype carriers, the rs118050317 CC genotype carriers were significant increased the endometrial cancer risk under co-dominant model (OR 8.43, 95% CI 1.05–67.89, *p* = 0.045), the results also significant under log-additive model (OR 1.47, 95% CI 1.04–2.07, *p* = 0.029) (Additional file 1: Table S2).

**Table 4:** Genotype frequencies of the SNPs and their associations with risk of endometrial cancer

SNP	Model	Genotype	Case	Control	Without adjustment		With adjustment of age	
					OR (95% CI)	*p*	OR (95% CI)	*p*
rs118050317	Co-dominant	GG	268	282	1		1	
		CG	74	61	1.28 (0.87–1.86)	0.206	1.28 (0.88–1.87)	0.202
		CC	8	1	8.42 (1.05–67.76)	0.045*	8.43 (1.05–67.89)	0.045*
	Dominant	GG	268	282	1		1	
		CG + CC	82	62	1.39 (0.96–2.01)	0.080	1.40 (0.96–2.02)	0.078
	Recessive	GG + CG	342	343	1		1	
		CC	8	1	8.02 (1.00–64.50)	0.050	8.03 (1.00–64.52)	0.050
	Log-additive	–	–	–	1.46 (1.04–2.06)	0.029*	1.47 (1.04–2.07)	0.029*

*OR* odds ratio, *95% CI* 95% confidential interval

*p < 0.05 indicates statistical significance

In the association analysis between different SNPs and different clinical index, we found there was significant difference between rs7307242 different genotype and CEA and AFP quantity (*p* = 0.021, *p* < 0.001), genotype AA corresponding to the highest quantity, followed by TT and AT genotype. rs75750647 different genotypes were significant associated with CA125 and CA199 quantity (*p* = 0.032, *p* = 0.033), the AA genotype carriers had the highest CA125 and CA199 quantity. SNP loci rs11610368 different genotypes were significant associated with HE4 quantity (*p* = 0.010), genotype AA corresponding to the highest quantity, followed by GG and AG (Table 5, Additional file 1: Table S1).

**Table 5:** The association between SNPs of *NINJ2* and clinical index of endometrial cancer

SNP	Clinical Index	Genotype	Number in cases	Quantity in blood (mean ± SD)	*p*
rs7307242	CEA (ng/ml)	AA	7	14.94 ± 3.97	0.021*
		AT	69	11.56 ± 3.55	
		TT	229	12.78 ± 4.01	
	AFP (ng/ml)	AA	7	19.55 ± 21.16	< 0.001*
		AT	69	9.23 ± 3.82	
		TT	227	9.61 ± 4.23	
rs75750647	CA125 (U/ml)	AA	35	46.51 ± 131.23	0.032*
		AG	129	24.09 ± 44.88	
		GG	135	18.6 ± 24.39	
	CA199 (U/ml)	AA	31	46.19 ± 163.92	0.033*
		AG	124	15.9 ± 14.45	
		GG	130	20.32 ± 28.95	
rs11610368	HE4 (pg/ml)	AA	3	295.38 ± 390.97	0.010*
		AG	65	90.37 ± 99.74	
		GG	233	91.49 ± 113.83	

*CEA* carcinoembryonic antigen, *CA* carbohydrate antigen, *HE4* human epididymis protein 4, *AFP* alpha fetoprotein, *SF* serum ferritin, *TNF* tumor necrosis factor

*p < 0.05 indicates statistical significance

## Discussion

In this study, we did an association study between *NINJ2* gene polymorphism and endometrial cancer risk in 351 endometrial cancer patients and 344 healthy controls. We found the SNP rs118050317 mutant allele C and homozygote CC genotype were significant increased the endometrial cancer risk. In the clinical index analysis, there was significant higher quantity of CEA, CA125 and AFP in cases than controls.

The gene encodes *NINJ2* is located on chromosome 12p13. Toshiyuki et al. showed *NINJ2* is a cell surface adhesion molecule [22]. As a vascular susceptibility gene, the SNPs in *NINJ2* were genotyped to test for the association between variants of *NINJ2* and dementia risk. It was found that the homozygosity of two SNPs rs11833579 and rs12425791 were associated with a decreased risk of Alzheimer’s disease [13]. Moreover, the researchers also found the SNP rs12425791 of *NINJ2* was significantly associated with ischemic stroke, and A allele increases the susceptibility of stroke [23]. In a family-based case control study, the A allele of rs11833579 may play a role in mediating susceptibility to ischemic stroke [14]. A functional polymorphism loci rs3809263 in the *NINJ2* promoter was significant decreased the large artery atherosclerotic stroke risk. Moreover, the AA genotype carriers had significantly increased *NINJ2* mRNA expression levels in Chinese population [15].

Previous study reported *NINJ1*, a homologue of *NINJ2*, can mediate inflammation processes [24]. In addition, *NINJ2* is expressed in lymphocyte cell. Inflammation was likely to increase risk of developing endometrial cancer [25]. Wang et al. found *NINJ2* can regulate the express of gene that associated with inflammation in human vascular endothelial cells, such as IL-6 [26]. In other study, a hypothesis about chronic inflammation may play an important role in endometrial carcinogenesis were investigated. The association between SNP in inflammatory pathway genes and endometrial cancer risk were conducted and found IL-6 SNP rs2069852 had an association with endometrial cancer risk [16]. So we speculate the rs118050317 C allele of *NINJ2* through regulate the IL-6 expression to influence the ERK-NF-κB signaling pathway of endometrial cancer growth [27]. The underlying mechanism need to be further investigated.

In this study, there were some limitations. First, endometrial cancer is a high incidence of female reproductive system tumors but relatively low incidence in the population. So the example size of our study was relatively small, we will collect as many samples as we can and verify our results in a larger population in future. Second, the clinical information were limited in this study, to enrich our study, we would continue to collect the information of patients and prognosis information to analyze the association between *NINJ2* gene and clinical information. The underlying mechanism of *NINJ2* gene involving in endometrial cancer need to be researched next step.

## Conclusion

In conclusion, the *NINJ2* gene polymorphism loci rs118050317 mutant allele C was associated with an increased risk of endometrial cancer and there was significant higher quantity in serum of CEA, CA125 and AFP in cases than controls.

## Supplementary information

**Additional file 1: Table S1.** The association between SNPs of *NINJ2* and clinical index of endometrial cancer**. Additional file 1: Table S2.** Genotype frequencies of the SNPs and their associations with risk of endometrial cancer

## Data Availability

All the data supporting the findings of this study are contained in the manuscript.

## Abbreviations

*NINJ2*: *Ninjurin2*
CEA: Carcinoembryonic antigen
CA125: Carbohydrate antigen 125
AFP: Alpha fetoprotein
HE4: Human epididymis protein 4
SF: Serum ferritin
TNF: Tumor necrosis factor
MAF: minor allele frequency
OR: Odds ratios
95% CI: 95% confidence interval
HWE: Hardy–weinberg equilibrium
SNP: Single nucleotide polymorphism
ERK: Extracellular signal-regulated kinase
