# Supplementary material for: Ninjurin 2 rs118050317 gene polymorphism and endometrial cancer risk
Source: Cancer Cell Int. 2021 Jan 4;21:1. doi: 10.1186/s12935-020-01646-5 (PMC7784262; doi:10.1186/s12935-020-01646-5)
Supplement: Supplementary file 1 — Additional file 1: Table S1. The association between SNPs of NINJ2 and clinical index of endometrial cancer. Additional file 1: Table S2. Genotype frequencies of the SNPs and their associations with risk of endometrial cancer [file 12935_2020_1646_MOESM1_ESM.docx]

**Additional file 1: Table S1**

The association between SNPs of *NINJ2* and clinical index of endometrial cancer

| Clinical Index | SNP | Genotype | Number in cases | Expression quantity  (Mean ± SD) | *p* |
| --- | --- | --- | --- | --- | --- |
| CEA (ng/ml) | rs118050317 | CC | 6 | 10.79 ± 2.90 | 0.502 |
|  |  | CG | 65 | 12.77 ± 3.59 |  |
|  |  | GG | 233 | 12.55 ± 4.07 |  |
|  | rs75750647 | AA | 35 | 12.14 ± 4.96 | 0.780 |
|  |  | AG | 132 | 12.54 ± 3.88 |  |
|  |  | GG | 138 | 12.67 ± 3.75 |  |
|  | rs7307242 | AA | 7 | 14.94 ± 3.97 | 0.021* |
|  |  | AT | 69 | 11.56 ± 3.55 |  |
|  |  | TT | 229 | 12.78 ± 4.01 |  |
|  | rs10849390 | GG | 43 | 12.86 ± 3.91 | 0.660 |
|  |  | GA | 138 | 12.67 ± 3.92 |  |
|  |  | AA | 124 | 12.31 ± 4.02 |  |
|  | rs11610368 | AA | 4 | 12.22 ± 2.56 | 0.711 |
|  |  | AG | 66 | 12.91 ± 4.30 |  |
|  |  | GG | 235 | 12.46 ± 3.88 |  |
| CA125 (U/ml) | rs118050317 | CC | 6 | 16.02 ± 13.24 | 0.705 |
|  |  | CG | 63 | 19.46 ± 21.97 |  |
|  |  | GG | 229 | 25.56 ± 63.16 |  |
|  | rs75750647 | AA | 35 | 46.51 ± 131.23 | 0.032* |
|  |  | AG | 129 | 24.09 ± 44.88 |  |
|  |  | GG | 135 | 18.6 ± 24.39 |  |
|  | rs7307242 | AA | 7 | 14.68 ± 6.08 | 0.869 |
|  |  | AT | 68 | 22.81 ± 59.76 |  |
|  |  | TT | 224 | 24.96 ± 56.20 |  |
|  | rs10849390 | GG | 42 | 21.10 ± 32.45 | 0.224 |
|  |  | GA | 135 | 19.11 ± 18.81 |  |
|  |  | AA | 122 | 30.98 ± 83.55 |  |
|  | rs11610368 | AA | 3 | 70.21 ± 93.91 | 0.266 |
|  |  | AG | 66 | 18.91 ± 18.56 |  |
|  |  | GG | 230 | 25.16 ± 62.56 |  |
| CA199 (U/ml) | rs118050317 | CC | 6 | 13.87 ± 13.33 | 0.914 |
|  |  | CG | 65 | 19.59 ± 29.07 |  |
|  |  | GG | 213 | 21.97 ± 65.41 |  |
|  | rs75750647 | AA | 31 | 46.19 ± 163.92 | 0.033* |
|  |  | AG | 124 | 15.9 ± 14.45 |  |
|  |  | GG | 130 | 20.32 ± 28.95 |  |
|  | rs7307242 | AA | 7 | 13.57 ± 9.17 | 0.937 |
|  |  | AT | 67 | 20.83 ± 30.08 |  |
|  |  | TT | 211 | 21.58 ± 65.54 |  |
|  | rs10849390 | GG | 40 | 17.23 ± 15.39 | 0.392 |
|  |  | GA | 131 | 17.38 ± 21.79 |  |
|  |  | AA | 114 | 27.00 ± 88.52 |  |
|  | rs11610368 | AA | 4 | 9.80 ± 9.69 | 0.690 |
|  |  | AG | 61 | 16.30 ± 13.3 |  |
|  |  | GG | 220 | 22.78 ± 65.84 |  |
| HE4 (pg/ml) | rs118050317 | CC | 6 | 94.9 ± 111.19 | 0.482 |
|  |  | CG | 65 | 77.82 ± 57.08 |  |
|  |  | GG | 229 | 97.67 ± 128.79 |  |
|  | rs75750647 | AA | 35 | 130.35 ± 188.84 | 0.134 |
|  |  | AG | 131 | 87.72 ± 82.74 |  |
|  |  | GG | 135 | 89.07 ± 119.55 |  |
|  | rs7307242 | AA | 7 | 80.99 ± 20.63 | 0.139 |
|  |  | AT | 68 | 69.21 ± 47.31 |  |
|  |  | TT | 226 | 100.91 ± 131.08 |  |
|  | rs10849390 | GG | 42 | 112.59 ± 176.29 | 0.358 |
|  |  | GA | 137 | 84.35 ± 91.27 |  |
|  |  | AA | 122 | 96.67 ± 116.31 |  |
|  | rs11610368 | AA | 3 | 295.38 ± 390.97 | 0.010* |
|  |  | AG | 65 | 90.37 ± 99.74 |  |
|  |  | GG | 233 | 91.49 ± 113.83 |  |
| AFP (ng/ml) | rs118050317 | CC | 6 | 9.09 ± 6.19 | 0.933 |
|  |  | CG | 64 | 9.66 ± 4.39 |  |
|  |  | GG | 232 | 9.81 ± 5.50 |  |
|  | rs75750647 | AA | 35 | 9.89 ± 4.32 | 0.537 |
|  |  | AG | 131 | 9.37 ± 4.28 |  |
|  |  | GG | 137 | 10.08 ± 6.28 |  |
|  | rs7307242 | AA | 7 | 19.55 ± 21.16 | <0.001* |
|  |  | AT | 69 | 9.23 ± 3.82 |  |
|  |  | TT | 227 | 9.61 ± 4.23 |  |
|  | rs10849390 | GG | 43 | 10.84 ± 4.60 | 0.083 |
|  |  | GA | 137 | 9.05 ± 4.11 |  |
|  |  | AA | 123 | 10.16 ± 6.45 |  |
|  | rs11610368 | AA | 4 | 12.29 ± 4.76 | 0.613 |
|  |  | AG | 66 | 9.84 ± 4.63 |  |
|  |  | GG | 233 | 9.68 ± 5.46 |  |
| SF (ng/ml) | rs118050317 | CC | 6 | 91.88 ± 8.56 | 0.496 |
|  |  | CG | 65 | 87.91 ± 8.64 |  |
|  |  | GG | 233 | 88.33 ± 7.63 |  |
|  | rs75750647 | AA | 35 | 85.74 ± 11.92 | 0.120 |
|  |  | AG | 132 | 88.52 ± 7.25 |  |
|  |  | GG | 138 | 88.73 ± 7.05 |  |
|  | rs7307242 | AA | 7 | 89.64 ± 7.85 | 0.807 |
|  |  | AT | 69 | 88.66 ± 6.55 |  |
|  |  | TT | 229 | 88.15 ± 8.23 |  |
|  | rs10849390 | GG | 43 | 89.64 ± 7.65 | 0.341 |
|  |  | GA | 138 | 87.69 ± 7.61 |  |
|  |  | AA | 124 | 88.51 ± 8.19 |  |
|  | rs11610368 | AA | 4 | 87.73 ± 8.49 | 0.940 |
|  |  | AG | 66 | 88.03 ± 7.46 |  |
|  |  | GG | 235 | 88.38 ± 7.99 |  |
| TNF (fmol/ml) | rs118050317 | CC | 6 | 0.89 ± 0.07 | 0.957 |
|  |  | CG | 65 | 0.89 ± 0.06 |  |
|  |  | GG | 233 | 0.89 ± 0.05 |  |
|  | rs75750647 | AA | 35 | 0.89 ± 0.06 | 0.701 |
|  |  | AG | 132 | 0.89 ± 0.05 |  |
|  |  | GG | 138 | 0.90 ± 0.06 |  |
|  | rs7307242 | AA | 7 | 0.92 ± 0.07 | 0.549 |
|  |  | AT | 69 | 0.89 ± 0.06 |  |
|  |  | TT | 229 | 0.89 ± 0.05 |  |
|  | rs10849390 | GG | 43 | 0.89 ± 0.06 | 0.771 |
|  |  | GA | 138 | 0.90 ± 0.05 |  |
|  |  | AA | 124 | 0.89 ± 0.06 |  |
|  | rs11610368 | AA | 4 | 0.86 ± 0.06 | 0.164 |
|  |  | AG | 66 | 0.90 ± 0.05 |  |
|  |  | GG | 235 | 0.89 ± 0.06 |  |

CEA: Carcinoembryonic antigen; CA: Carbohydrate antigen; HE4: Human epididymis protein 4; AFP: Alpha fetoprotein; SF: Serum ferritin; TNF: Tumor necrosis factor

**p <*0.05 indicates statistical significance

**Additional file 1: Table S2**

Genotype frequencies of the SNPs and their associations with risk of endometrial cancer

| SNP | Model | Genotype | Case | Control | Without adjustment | | With adjustment of age | |
| --- | --- | --- | --- | --- | --- | --- | --- | --- |
|  |  |  |  |  | OR (95% CI) | *p* | OR (95% CI) | *p* |
| rs118050317 | Co-dominant | GG | 268 | 282 | 1 |  | 1 |  |
|  |  | CG | 74 | 61 | 1.28 (0.87 - 1.86) | 0.206 | 1.28 (0.88 - 1.87) | 0.202 |
|  |  | CC | 8 | 1 | 8.42 (1.05 - 67.76) | 0.045* | 8.43 (1.05 - 67.89) | 0.045* |
|  | Dominant | GG | 268 | 282 | 1 |  | 1 |  |
|  |  | CG+CC | 82 | 62 | 1.39 (0.96 - 2.01) | 0.080 | 1.40 (0.96 - 2.02) | 0.078 |
|  | Recessive | GG+CG | 342 | 343 | 1 |  | 1 |  |
|  |  | CC | 8 | 1 | 8.02 (1.00 - 64.50) | 0.050 | 8.03 (1.00 - 64.52) | 0.050 |
|  | Log-additive | — | — | — | 1.46 (1.04 - 2.06) | 0.029* | 1.47 (1.04 - 2.07) | 0.029* |
| rs75750647 | Co-dominant | GG | 158 | 164 | 1 |  | 1 |  |
|  |  | AG | 152 | 144 | 1.10 (0.80 - 1.50) | 0.571 | 1.10 (0.80 - 1.50) | 0.573 |
|  |  | AA | 41 | 36 | 1.18 (0.72 - 1.95) | 0.510 | 1.18 (0.72 - 1.95) | 0.513 |
|  | Dominant | GG | 158 | 164 | 1 |  | 1 |  |
|  |  | AG+AA | 193 | 180 | 1.11 (0.83 - 1.50) | 0.482 | 1.11 (0.83 - 1.50) | 0.485 |
|  | Recessive | GG+AG | 310 | 308 | 1 |  | 1 |  |
|  |  | AA | 41 | 36 | 1.13 (0.70 - 1.82) | 0.610 | 1.13 (0.70 - 1.82) | 0.613 |
|  | Log-additive | — | — | — | 1.09 (0.87 - 1.36) | 0.446 | 1.09 (0.87 - 1.36) | 0.449 |
| rs7307242 | Co-dominant | TT | 262 | 245 | 1 |  | 1 |  |
|  |  | AT | 81 | 94 | 0.81 (0.57 - 1.14) | 0.219 | 0.81 (0.57 - 1.14) | 0.220 |
|  |  | AA | 8 | 4 | 1.87 (0.56 - 6.29) | 0.312 | 1.87 (0.56 - 6.29) | 0.312 |
|  | Dominant | TT | 262 | 245 | 1 |  | 1 |  |
|  |  | AT+AA | 89 | 98 | 0.85 (0.61 - 1.19) | 0.340 | 0.85 (0.61 - 1.19) | 0.342 |
|  | Recessive | TT+AT | 343 | 339 | 1 |  | 1 |  |
|  |  | AA | 8 | 4 | 1.98 (0.59 - 6.63) | 0.270 | 1.98 (0.59 - 6.62) | 0.270 |
|  | Log-additive | — | — | — | 0.92 (0.68 - 1.24) | 0.571 | 0.92 (0.68 - 1.24) | 0.574 |
| rs10849390 | Co-dominant | AA | 143 | 141 | 1 |  | 1 |  |
|  |  | GA | 155 | 152 | 1.01 (0.73 - 1.39) | 0.974 | 1.01 (0.73 - 1.39) | 0.974 |
|  |  | GG | 53 | 46 | 1.14 (0.72 - 1.80) | 0.585 | 1.14 (0.72 - 1.80) | 0.580 |
|  | Dominant | AA | 143 | 141 | 1 |  | 1 |  |
|  |  | GA+GG | 208 | 198 | 1.04 (0.76 - 1.40) | 0.820 | 1.04 (0.77 - 1.40) | 0.818 |
|  | Recessive | AA+GA | 298 | 293 | 1 |  | 1 |  |
|  |  | GG | 53 | 46 | 1.13 (0.74 - 1.74) | 0.567 | 1.14 (0.74 - 1.74) | 0.561 |
|  | Log-additive | — | — | — | 1.05 (0.85 - 1.30) | 0.653 | 1.05 (0.85 - 1.30) | 0.648 |
| rs11610368 | Co-dominant | GG | 268 | 264 | 1 |  | 1 |  |
|  |  | AG | 77 | 69 | 1.10 (0.76 - 1.59) | 0.613 | 1.10 (0.76 - 1.59) | 0.614 |
|  |  | AA | 6 | 10 | 0.59 (0.21 - 1.65) | 0.315 | 0.59 (0.21 - 1.65) | 0.316 |
|  | Dominant | GG | 268 | 264 | 1 |  | 1 |  |
|  |  | AG+AA | 83 | 79 | 1.04 (0.73 - 1.47) | 0.848 | 1.04 (0.73 - 1.47) | 0.849 |
|  | Recessive | GG+AG | 345 | 333 | 1 |  | 1 |  |
|  |  | AA | 6 | 10 | 0.58 (0.21 - 1.61) | 0.295 | 0.58 (0.21 - 1.61) | 0.296 |
|  | Log-additive | — | — | — | 0.98 (0.72 - 1.32) | 0.873 | 0.98 (0.72 - 1.32) | 0.873 |

OR: odds ratio; 95% CI: 95% confidential interval

**p <*0.05 indicates statistical significance
